# Enhancing the Ecological Validity of fMRI Memory Research Using Virtual Reality

**DOI:** 10.3389/fnins.2018.00408

**Published:** 2018-06-15

**Authors:** Nicco Reggente, Joey K.-Y. Essoe, Zahra M. Aghajan, Amir V. Tavakoli, Joseph F. McGuire, Nanthia A. Suthana, Jesse Rissman

**Affiliations:** ^1^Department of Psychology, University of California, Los Angeles, Los Angeles, CA, United States; ^2^Department of Psychiatry & Biobehavioral Sciences, University of California, Los Angeles, Los Angeles, CA, United States; ^3^Division of Biology and Biological Engineering, California Institute of Technology, Pasadena, CA, United States; ^4^Department of Neurosurgery, University of California, Los Angeles, Los Angeles, CA, United States; ^5^Division of Child and Adolescent Psychiatry, Johns Hopkins Children’s Center, Johns Hopkins Medicine, Baltimore, MD, United States

**Keywords:** functional magnetic resonance imaging (fMRI), virtual reality (VR), memory, ecological validity, context

## Abstract

Functional magnetic resonance imaging (fMRI) is a powerful research tool to understand the neural underpinnings of human memory. However, as memory is known to be context-dependent, differences in contexts between naturalistic settings and the MRI scanner environment may potentially confound neuroimaging findings. Virtual reality (VR) provides a unique opportunity to mitigate this issue by allowing memories to be formed and/or retrieved within immersive, navigable, visuospatial contexts. This can enhance the ecological validity of task paradigms, while still ensuring that researchers maintain experimental control over critical aspects of the learning and testing experience. This mini-review surveys the growing body of fMRI studies that have incorporated VR to address critical questions about human memory. These studies have adopted a variety of approaches, including presenting research participants with VR experiences in the scanner, asking participants to retrieve information that they had previously acquired in a VR environment, or identifying neural correlates of behavioral metrics obtained through VR-based tasks performed outside the scanner. Although most such studies to date have focused on spatial or navigational memory, we also discuss the promise of VR in aiding other areas of memory research and facilitating research into clinical disorders.

## Introduction

**FIGURE 1:**
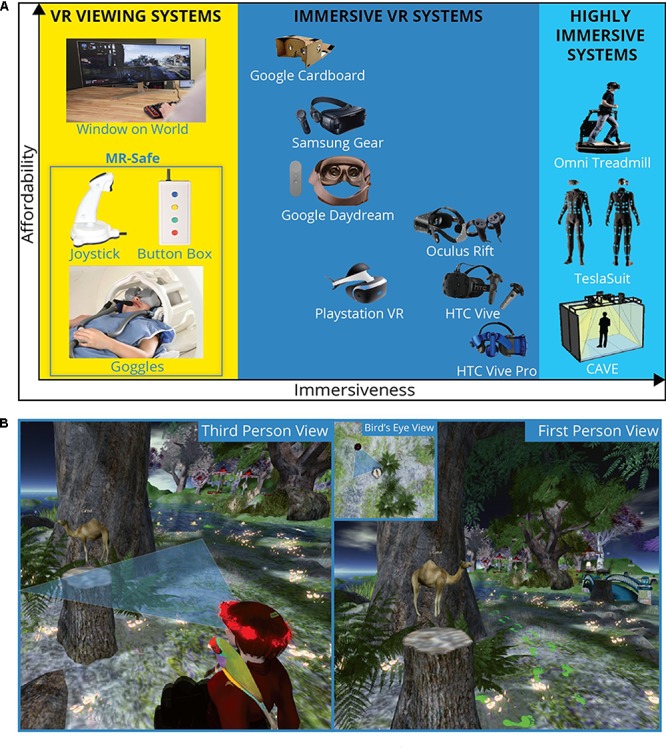
**(A)** A limited showcase of currently available VR technologies. Devices are sorted as a function of their ability to provide the participant with a sense that they are “in” a virtual environment (immersiveness; *x*-axis) and the system’s affordability (*y*-axis). “Window on World” refers to a traditional desktop and monitor setup. CAVE = cave automatic virtual environment – a real world room that leverages projectors and motion capture to create room-size virtual experiences. MR-safe equipment (joystick and buttonbox: Current Design, Inc., Philadelphia, PA, United States, goggles: cinemavision.biz) can be used during MR scans. **(B)** Examples of common perspectives presented to participants while actively navigating VEs or during spatial memory tests. Both first- and third-person viewpoints provide an egocentric perspective whereas a bird’s eye view provides an allocentric one.

Virtual reality (VR) is a term used to encompass any computer-generated experience that induces a sense of presence – the feeling of being transported to and inhabiting a place different from one’s immediate surroundings (Steuer, 1992; McCreery et al., 2013). Given the intimate relationship between context and memory (Godden and Baddeley, 1975; Smith, 1988; Ramirez et al., 2013), VR offers a powerful means to enhance the ecological validity of memory research by providing realistic virtual environments (VEs) in which participants can learn information and/or draw upon past memories to guide their behavior. These VEs can be highly customized to meet the needs of a wide variety of tasks and offer experimental control over the learning experience. Given these characteristics, along with the recent surge in VR technological development and accessibility (**Figure 1A**), it is unsurprising that cognitive neuroscientists interested in the brain mechanisms of memory have increasingly found ways to incorporate VR into their fMRI studies.

**FIGURE 2:**
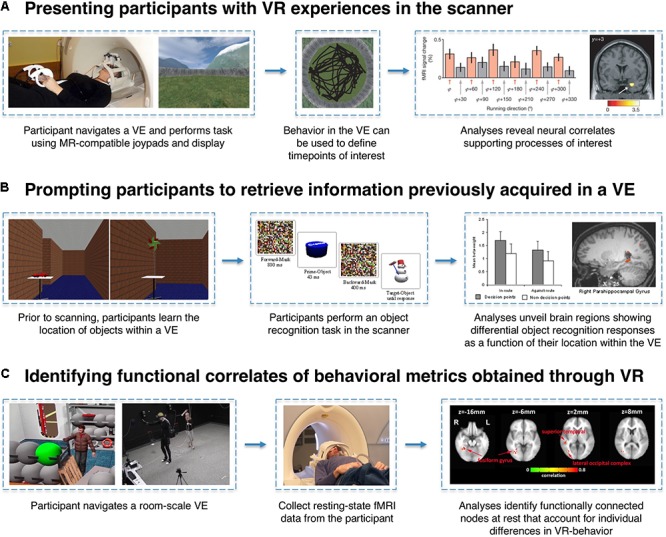
Examples of VR-fMRI experimental paradigms. **(A)** MR-compatible joysticks/gamepads and 3D stereoscopic goggles allow for participants to enter a VE while laying supine in the scanner. By time-locking events of interest (e.g., a participant’s heading direction while traversing the world) to the corresponding fMRI signals, researchers can identify neural correlates associated with specific task conditions or behaviors. In this example, entorhinal cortex activity is associated with the grid-cell-like property of hexagonal symmetry during navigation. Figures adapted with permission from Doeller et al. (2010). **(B)** Participants can perform VR-based learning tasks outside of the scanner, and their memory for information encoded within a VE can later be tested in the scanner using traditional fMRI task paradigms. In this example, trials can be coded based on each object’s properties within the VE (e.g., whether or not the object was located at a pertinent decision-point) to reveal incidental neural differences during retrieval as a function of the encoding experience. Figures adapted with permission from Janzen and Weststeijn (2007). **(C)** Just as questionnaires and computer tasks reveal individual differences in a host of behavioral metrics, VR can serve as an instrument to gather unique behavioral data points (e.g., number of times a participant revisited a particular location). Researchers can then examine whether these performance metrics can account for variance in brain activity or connectivity measured in a completely different context (e.g., while participants are simply resting in the scanner). Figures adapted with permission from Wong et al. (2014).

Experimental designs employing VR and fMRI to study memory predominantly fall into three categories: (1) having participants actively engage in VR experiences in the scanner while functional neuroimaging data are acquired, (2) scanning participants as they are prompted to retrieve information previously acquired in a VE, and (3) identifying structural or functional correlates of behavioral metrics obtained through the use of VR (**Figure 2**). One virtue of VR as an experimental tool is its ability to enable the translation of research paradigms that have been used extensively in animal research, which may not otherwise translate readily to human participants for ethical or technical reasons. For example, a direct human analog of the Morris water maze – dropping a participant into a pool of cloudy water in search of an invisible platform – would likely raise the ethical eyebrows of any Institutional Review Board, yet such a task paradigm can be implemented in VR. Likewise, VR empowers neuroscientists to create experiments that would either be impossible or impractical without the use of VR (e.g., imposing invisible boundaries, altering/morphing environmental features, or teleporting a participant between contexts).

Researchers may go to great lengths to increase the ecological validity of their tasks, given the growing appreciation that laboratory-encoded stimuli and real-world events tend to evoke different brain activation profiles (Roediger and McDermott, 2013; Chen et al., 2017; Chow et al., 2018). For instance, wearable cameras can be used to capture photographs of participants’ real-world experiences so that memories for these events can later be probed in the scanner (Chow and Rissman, 2017). A related approach involves having participants engage in real-world navigation tasks. In one such study, Schinazi and Epstein (2010) created a 3-km outdoor walking course for participants to traverse. Later, fMRI data were collected while participants were tested on their recollection for buildings encountered on the route. While the fMRI results revealed interesting effects within visuospatial processing regions such as the retrosplenial cortex, reflecting the interplay between landmark-identification and route direction at navigationally pertinent decision points, the authors acknowledged that their behavioral results were largely consistent with those of a similarly designed VR study by Janzen and Weststeijn (2007). A subsequent fMRI study then showed that comparable neuroimaging findings could be obtained used a VR-based route navigation task (Wegman and Janzen, 2011). Although real-world task paradigms will continue to have value in memory research, VR paradigms have the potential to provide a less labor-intensive and more highly controlled investigational medium that sacrifices relatively little in terms of neural processing and experimental outcome.

While VR allows for precise control over stimuli and contexts, providing greater consistency across participants than can typically be attained in real-world designs, it is not without its caveats. Recently, there has been debate as to whether VR-based navigation should be considered true navigation (Taube et al., 2013; Minderer et al., 2016). One of the most crucial arguments against the fusion of VR and fMRI is that when lying in a scanner, vestibular self-motion (idiothetic) cues cannot match external landmark-based (allothetic) cues since otolith organs will persistently relay a signal that the individual is supine. Decoupling of cues can cause a reorientation (Wang and Spelke, 2002) and force one system into domination (Golledge, 1998; Dolins and Mitchell, 2010). Further adding to these complications, visual cues alone have proven insufficient to elicit accurate distance measurements (Witmer and Kline, 1998) and turn responses (Riecke et al., 2012), which can lead to impaired navigation. Meanwhile, on a neuronal level, the activity pattern of cells implicated in spatial representation, such as place cells, grid cells, and head-direction cells (Buzsáki and Moser, 2013) have been shown to differ between real-world environments and VEs (Chen et al., 2013; Ravassard et al., 2013; Aghajan et al., 2015).

Nevertheless, the neural responses of spatially selective cells in VR resemble those observed in real navigation under certain circumstances (Domnisoru et al., 2013; Aronov and Tank, 2014; Killian and Buffalo, 2018). Additionally, VR navigation has been shown to maintain hippocampal theta rhythms (Ekstrom et al., 2005), albeit with some differences from real-world navigation (Jacobs, 2014; Aghajan et al., 2017; Bohbot et al., 2017). Various VR accessories, including head-mounted displays (HMD) can be used to increase participants’ immersion (**Figure 1A**; Dede, 2009) and, subsequently, spatial understanding (Ruddle et al., 1997; Bowman and McMahan, 2007). Importantly, Ganesh et al. (2012) found that increasing participants’ self-identification with an avatar resulted in increased engagement of left inferior parietal lobe regions associated with self-identification and improved recognition memory for traits associated with their avatar. Furthermore, brain activity patterns expressed during recall remain similar despite encoding in real-world vs. fictional environments (Spiers and Maguire, 2006). Even navigation through digital folders (Benn et al., 2015) and abstract conceptual space (Constantinescu et al., 2016) recruits similar brain structures and processes.

Given that the overarching goal of cognitive neuroscience research is to understand the brain mechanisms that give rise to our thoughts and behaviors, VR affords researchers with the ability to execute task paradigms that more closely mimic the way we use our cognition as we dynamically engage with our environment. This mini-review surveys the burgeoning neuroimaging literature on VR applications to memory research. In so doing, we hope to illustrate some creative ways in which researchers have leveraged VR to increase the ecological validity of memory experiments and conduct studies that would be relatively infeasible without the use of VR.

## Harnessing the Affordances of VR to Aid Memory Research

Although neural recordings from freely moving rodents have provided crucial insights into spatial memory functioning, ethical and physical limitations have prevented a direct replication of these studies in human participants. However, VR offers researchers boundless, safe, and controllable environments to conduct analogs of foundational experimental paradigms like the Morris water maze (MWM; Morris, 1984), radial arm maze (RAM; Olton et al., 1977), and random foraging tasks. Indeed, when combined with fMRI, VR has afforded researchers with the ability to quickly iterate manipulations of different MWM task features (e.g., distal vs. no cues; visible vs. invisible platforms) to determine hippocampal dependence (Shipman and Astur, 2008; Kolarik et al., 2016), identify compensatory mechanisms following scopolamine injection (Antonova et al., 2011), examine functional connectivity changes (Woolley et al., 2015), and investigate the different neural patterns recruited when using egocentric vs. allocentric navigation strategies (Rodriguez, 2010a). A research group even recently replicated their rodent body-behavior findings in humans using a VR version of the MWM (Müller et al., 2018).

Virtual variations of the RAM have equipped researchers to study working memory and decision-making in both win-shift (Demanuele et al., 2015) and win-stay (Cyr et al., 2016) paradigms. VR also allows for real-time changes to RAM and similar tasks. For instance, shuffling distal cues and providing visual navigational guidance (e.g., following arrows on the ground) has made it possible to disentangle cognitive decision-making from other processes of interest (Marsh et al., 2010). The ability to “teleport”, restrict access to certain areas with virtual “walls”, and track the precise location of the subject within the VE permit researchers to tease apart place-based and sequence-based strategies (Igloi et al., 2015). VR versions of the RAM were also used to assess the integrity of the hippocampus – predicting risk or severity in a variety of psychiatric disorders (Astur et al., 2005; Wilkins et al., 2017). Such insights are in line with the growing trend of using VR to provide objective diagnostic metrics (Cogné et al., 2017; van Bennekom et al., 2017). For instance, Migo et al. (2016) identified behavioral and neural correlates of completing the RAM task in patients with amnestic mild cognitive impairment (MCI), which extends upon the work of King et al. (2002) who showed that when changing virtual viewpoints, MCI patients could not recall the positions of objects. Similar spatial memory tests have been conducted on athletes following mild traumatic brain injury (Slobounov et al., 2010).

Given the expanse of possibilities afforded by VR, experimental paradigms can move beyond the replication of rodent studies. By familiarizing participants with a VE, experimenters can probe a participant’s spatial memory by asking them to navigate from one location to another – a general paradigm that also can be used to test orientation, route-learning, and viewpoint-dependence (Brown et al., 2014; Stokes et al., 2015; Dimsdale-Zucker et al., 2018). Indeed, many such studies have used VEs to examine the neural correlates supporting navigation under different manipulations such as: using one landmark vs. many (Wegman et al., 2014), finding one’s way vs. following a visible path (Hartley et al., 2003), relying on coarse vs. global strategies (Evensmoen et al., 2013), leveraging survey vs. route knowledge (Gillner and Mallot, 1998; Wolbers et al., 2004), tracking paths and distances (Wolbers et al., 2007; Chrastil et al., 2015), varying head directions (Shine et al., 2016), egocentric and/or allocentric related manipulations (Wolbers et al., 2008; Suthana et al., 2009), and navigating towards a goal in healthy (Rodriguez, 2010b; Brown et al., 2016) and clinical populations (Thomas et al., 2001). Embedding several such manipulations within a single VR study, Dhindsa et al. (2014) utilized fMRI to measure signal fluctuations as participants oriented themselves towards a learned location in a VE that lost critical features one-by-one. Their results provided empirical evidence in support of the Byrne et al. (2007) model of orientation and navigation, which emphasizes the translation of egocentric representations in parietal cortex to allocentric representations in the hippocampus. Furthermore, virtual renditions of familiarized real-world environments can allow researchers to probe memory for real-world objects using virtual cues – a technique previously used to examine the neural correlates of egocentric representations for objects outside of one’s visual field (Schindler and Bartels, 2013).

The use of concurrent fMRI and VR also begets an opportunity to examine the neural underpinnings of spatial information that is being encoded incidentally. For example, following periods of egocentric navigation, researchers can provide participants with a spatial memory test using a bird’s eye view of the environment (**Figure 1B**) – a metric of allocentric memory that has been used to explain differences in navigational ability (Pine et al., 2002). Other examples come from fMRI studies looking for evidence of pattern separation and pattern completion processes (Yassa and Stark, 2011). By having participants complete the same relative distance task across different, but visually similar, environments, Kyle et al. (2015) found that the more distinguishable a neural representation is of an environment (i.e., successful pattern separation), the less the interference of competing memories will hinder performance. Relatedly, a human analog of the attractor dynamic model of mnemonic processing (Leutgeb et al., 2007) was demonstrated by Steemers et al. (2016): hippocampal responses to VEs that were constructed by linearly morphing two previously known VEs exhibited non-linear (sigmoid-like) response properties indicative of pattern completion, despite participants’ behavioral reports that they consciously perceived linear morphs. By leveraging multivoxel pattern analysis in the hippocampus to decode a participant’s location within a virtual environment, Hassabis et al. (2009) corroborated the classic function of hippocampal place cells (O’Keefe and Dostrovsky, 1971), albeit at a far less granular level. VR-based random foraging tasks have also been used to identify population-based grid-cell-like activity patterns in human entorhinal cortex (Doeller et al., 2010) – a measurement whose consistency over time could be prognostic of Alzheimer’s Disease risk (Kunz et al., 2015) – and 3D place coding representations in the human hippocampus (Kim et al., 2017).

VEs can also be utilized to systematically, and quantitatively, investigate processes that rely on imagined navigation. For example, Legge et al. (2012) familiarized participants with a VE that they were later instructed to use as a “memory palace” while they implemented the Method of Loci mnemonic strategy of mentally “placing” a set of to-be-remembered items along a route within an imagined environment. In this way, the authors matched the size, detail, and exposure time to the environment – properties that are often confounded in traditional implementations of this mnemonic technique (Yates, 1966). Further, the use of imagined virtual navigation has revealed fMRI signals that exhibit grid-cell-like properties (Bellmund et al., 2016; Horner et al., 2016) and activity patterns associated with location and facing direction (Marchette et al., 2014). Equalizing environments used for imagination tasks is particularly relevant in the domain of prospective memory (the ability to maintain a representation of intended tasks and execute them at the appropriate time and place). For instance, VR has recently been used in conjunction with high-resolution fMRI to index the degree to which specific goal and sub-goal locations are represented within hippocampal activity patterns during route planning, reflecting prospective coding of navigational intentions (Brown et al., 2016). Additionally, Kalpouzos and Eriksson (2013) familiarized participants to a VE and subsequently collected fMRI data while they mentally executed intended tasks within the imagined VE – a design that reduced variability in neural representation for environment.

Given that a time-course of fMRI activity can be collected during virtual navigation, it is possible to examine the different temporal phases of navigation behavior (Demanuele et al., 2015). Previous work has examined: planning vs. execution (Xu et al., 2010), encoding vs. retrieval (Suthana et al., 2011), periods of object manipulation (Baumann et al., 2003a), and active vs. guided periods (Baumann et al., 2003b). Persson et al. (2013) measured hippocampal activity as participants navigated through a virtual maze and found that males and females show dissociable recruitment of left and right hippocampus during active navigation relative to orientation judgments made at maze end-points. Additionally, events that occur within VR (e.g., encountering another avatar who dispenses objects) can be dissociated from their visual scene context by using different approach routes (Burgess et al., 2001). Even metrics like memory for heading direction (Baumann and Mattingley, 2010) and environmental size/complexity (Baumann and Mattingley, 2013) can be investigated by examining fMRI activity levels at relevant task time points (e.g., when the participant is facing North; **Figure 2A**), without explicitly probing the participant.

In addition to navigation studies, VEs can be employed to study object-place associative memory. VR can be used to efficiently change the constellation of objects and their identities, with respect to locations within the VEs [e.g., shuffling object identities (Wong et al., 2014), modulating their saliency (Buchy et al., 2014), or altering the environment boundaries (Lee et al., 2016)]. Object-place memory tasks have also shown that emotion is bound to places by examining how the co-occurrence of task-irrelevant emotional events alongside encoding can heighten subsequent retrieval activity(Chan et al., 2014) – extending findings that show place cells remapping once an environment becomes associated with fear (Moita et al., 2004). VR allows for object-place experiments to be conducted with high precision, immersion, and repeatability – a set of capabilities that make it particularly useful for obtaining diagnostic metrics in clinical populations (e.g., schizophrenia patients; Hawco et al., 2015).

Performance on VR-based tasks can also serve as a useful measuring instrument for examining factors outside of the learning experience that may affect behavior. For instance, Rauchs et al. (2008) investigated the neurocognitive effects of sleep deprivation on a series of virtual navigation tests. Researchers can also examine how fMRI signals measured in one setting (e.g., during resting fixation) might predict individual differences in performance on VR-based tasks performed outside the scanner. For example, Wong et al. (2014) identified patterns of resting-state activity and functional connectivity that correlated with participants’ memory for objects that had been learned in a room-scale VE the day before. In another study, Wegman and Janzen (2011) scanned participants while passively viewing a route through a VE to identify brain regions associated with navigation-based decision points, later using the functional connectivity profile of those regions during resting-state to account for individual differences in spatial memory.

## Discussion

While fMRI has served as a powerful tool in human memory research, it requires participants to be placed in a context that is far from naturalistic – a potential confound for many memory studies. The inclusion of VR in fMRI memory investigations allows researchers to utilize immersive and navigable contexts for stimulus presentation both inside and outside the scanner (**Figure 1A**). Moreover, it affords researchers a medium in which to conduct experiments that is both replicable and controllable.

Facets unique to VR position it as an indispensable toolkit for specific types of investigations. For instance, creating invisible walls that restrict movement, but retain the visibility of distal cues would not be feasible outside of a VE (Lee et al., 2016). Work by Bergouignan et al. (2014), which used VR to induce out-of-body experiences in the scanner while examining the role of perceiving the world from the perspective of one’s own body for successful episodic encoding of real-life events, would not have been possible without the use of VR. The same concept applies to VR’s ability to “blend” VEs (Steemers et al., 2016) or shift participants’ perspective within the same VE (Sulpizio et al., 2016). Additionally, VR has the capacity to even the playing field in experiments that hinge on the use of imagination (e.g., Legge et al., 2012): it provides a common virtual space instead of relying on familiar real-world environments that could vary across individuals as a function of their pre-experimental exposure to the environment.

VR technologies can also bolster the ecological validity of fMRI for researchers and clinicians to obtain objective diagnostic metrics for patient populations (King et al., 2002; Plancher et al., 2012; Cogné et al., 2017; van Bennekom et al., 2017). With HMDs, cross-institutional collaboration can be facilitated as participants immersed in VR will not be cognizant of the real-world environmental cues. Such attributes are particularly advantageous for the examination of disorders that are highly context-dependent (e.g., post-traumatic stress disorder). For instance, researchers have utilized VR to induce context-specific fear-conditioning (Huff et al., 2011; Tröger et al., 2012; Ewald et al., 2014) and fear extinction (Dunsmoor et al., 2014; Ahs et al., 2015) – dramatically extending current treatment methods which often require therapy to occur in a context that is dissimilar from where the fear was acquired (for review see Bohil et al., 2011; Maples-Keller et al., 2017). Furthermore, compared to many real-world tasks, VR-based experimental techniques can be replicated in shorter time spans.

The utilization of VR in fMRI studies need not be daunting nor expensive; open-source software such as OpenSimulator^1^ and equipment found in most scanner suites (**Figure 1A**), such as MR-compatible stereoscopic goggles and joysticks/joypads, make it increasingly accessible. Nonetheless, VR research is still in its infancy and not without limitations. Given the visual–vestibular disconnect of most setups, some participants may experience nausea and be unable to complete the study (Sharples et al., 2008). However, advances in HMD technology are already helping to alleviate motion-sickness concerns. Devices that increase immersion through haptic feedback (e.g., Tesla Suit) and stationary locomotion (e.g., Omni Treadmill) or setups that create room-scale environments (e.g., cave automatic virtual environment; **Figure 1A**) afford researchers with the ability to employ encoding paradigms that increasingly resemble “real life” circumstances, making the neural correlates associated with the formation and recall of such memories more likely to generalize to real-world behaviors.

## Author Contributions

NR, JE, ZA, AT, JM, NS, and JR conducted the literature review and wrote the manuscript. AT, NR, and JR generated figures.

## Conflict of Interest Statement

The authors declare that the research was conducted in the absence of any commercial or financial relationships that could be construed as a potential conflict of interest.

## Footnotes

1 http://opensimulator.org
